# Supplementary material for: Computational prediction of drug response in short QT syndrome type 1 based on measurements of compound effect in stem cell-derived cardiomyocytes
Source: PLoS Comput Biol. 2021 Feb 16;17(2):e1008089. doi: 10.1371/journal.pcbi.1008089 (PMC7909705; doi:10.1371/journal.pcbi.1008089)
Supplement: S1 Appendix — (PDF) [file pcbi.1008089.s001.pdf]

Base model formulation

In this appendix, we describe the full base model formulation. Here, the membrane potential (v) is given in units of mV, and the Ca^{2+} and Na^{+} concentrations are given in units of mM. All currents are given in units of A/F, and the ionic fluxes are expressed as mmol/ms per total cell volume (i.e., in units of mM/ms). Time is given in ms. The parameters of the model are all given in Tables I–VII.

Note that this model formulation is largely based on the base model from [1]. The differences between the model specified here and the model from [1] are that the below model includes a model for changes in the intracellular Na^{+} concentration and that the formulation of the I_{Kr} current has been adjusted. In addition, the model has been extended to include temperature dependent changes in the conductances of the currents (in the form of Q_{10}-values) because we consider different temperatures for hiPSC-CMs (room temperature) and adult cells (body temperature).

The membrane potential

In the base model formulation, the membrane potential is governed by

$$\begin{aligned}\frac{dv}{dt} = -(&I_{\mathrm{Na}} + I_{\mathrm{NaL}} + I_{\mathrm{CaL}} + I_{\mathrm{to}} + I_{\mathrm{Kr}} + I_{\mathrm{Ks}} + I_{\mathrm{K1}} \\ &+ I_{\mathrm{NaCa}} + I_{\mathrm{NaK}} + I_{\mathrm{pCa}} + I_{\mathrm{bCl}} + I_{\mathrm{bCa}} + I_{\mathrm{f}} + I_{\mathrm{stim}}),\end{aligned} \tag{1}$$

where I_{Na}, I_{NaL}, I_{CaL}, I_{to}, I_{Kr}, I_{Ks}, I_{K1}, I_{NaCa}, I_{NaK}, I_{pCa}, I_{bCl}, I_{bCa}, and I_{f} are membrane currents that will be specified below and I_{stim} is an applied stimulus current. In our computations, we let I_{stim} be given as a constant current of size -5 A/F for hiPSC-CMs and -40 A/F for adult cells. The I_{stim} current is applied until the membrane potential reaches a value of -40 mV.

Membrane currents

In general, the currents through the voltage-gated ion channels on the cell membrane are given on the form

$$I = go(v - E),$$

where g is the channel conductance, v is the membrane potential and E is the equilibrium potential of the channel. Moreover, o is the open probability of the channels, which is given on the form $o = \prod_i z_i$, where z_i are gating variables. These gating variables are either given as an explicit function of the membrane potential or governed by equations of the form

$$z_i' = \frac{1}{\tau_{z_i}}(z_{i,\infty} - z_i). \tag{2}$$

The parameters τ_{z_i} and $z_{i,\infty}$ are specified for each of the gating variables of the model in Table VIII.

Fast sodium current The formulation of the fast sodium current is based on the model formulation given in [2], adjusted to support slower upstroke velocities more similar to those observed for hiPSC-CMs. The current is given by

$$I_{\mathrm{Na}} = g_{\mathrm{Na}} o_{\mathrm{Na}}(v - E_{\mathrm{Na}}), \tag{3}$$

where the open probability is given by

$$o_{\mathrm{Na}} = m^3 j, \tag{4}$$

and m and j are gating variables governed by equations of the form (2).

Late sodium current The formulation of the late sodium current, I_{NaL}, is based on [3] and is given by

$$I_{\mathrm{NaL}} = g_{\mathrm{NaL}} o_{\mathrm{NaL}}(v - E_{\mathrm{Na}}), \tag{5}$$

where the open probability is given by

$$o_{\mathrm{NaL}} = m_L h_L, \tag{6}$$

and m_L and h_L are gating variables governed by equations of the form (2).

Transient outward potassium current The formulation of the transient outward potassium current, I_{to}, is based on [4] and is given by

$$I_{\mathrm{to}} = g_{\mathrm{to}} o_{\mathrm{to}} (v - E_{\mathrm{to}}), \tag{7}$$

where the open probability is given by

$$o_{\mathrm{to}} = q_{\mathrm{to}} r_{\mathrm{to}}, \tag{8}$$

and q_{to} and r_{to} are gating variables governed by equations of the form (2).

Rapidly activating potassium current The formulation of the rapidly activating potassium current, I_{Kr}, is based on [4], but is adjusted to measurements of steady state I_{Kr} currents for wild type and SQT from [5]. The current is given by

$$I_{\mathrm{Kr}} = g_{\mathrm{Kr}} o_{\mathrm{Kr}} (v - E_{\mathrm{K}}), \tag{9}$$

where

$$o_{\mathrm{Kr}} = x_{\mathrm{Kr1}} x_{\mathrm{Kr2}}, \tag{10}$$

and the dynamics of x_{Kr1} and x_{Kr2} are governed by equations of the form (2).

Slowly activating potassium current The formulation of the slowly activating potassium current, I_{Ks}, is based on [2] and is given by

$$I_{\mathrm{Ks}} = g_{\mathrm{Ks}} o_{\mathrm{Ks}} (v - E_{\mathrm{Ks}}), \tag{11}$$

where

$$o_{\mathrm{Ks}} = x_{\mathrm{Ks}}^2, \tag{12}$$

and the dynamics of x_{Ks} is governed by an equation of the form (2).

Inward rectifier potassium current The formulation of the inward rectifier potassium current, I_{K1}, is based on [2] and is given by

$$I_{\mathrm{K1}} = g_{\mathrm{K1}} o_{\mathrm{K1}} (v - E_{\mathrm{K}}), \tag{13}$$

where o_{K1} is given by the explicit formulas

$$o_{\mathrm{K1}} = \frac{a_{\mathrm{K1}}}{a_{\mathrm{K1}} + b_{\mathrm{K1}}}, \tag{14}$$

$$a_{\mathrm{K1}} = \frac{1}{1 + e^{0.2(v - E_K - 59)}}, \tag{15}$$

$$b_{\mathrm{K1}} = \frac{0.5 e^{0.08(v - E_K + 5)} + e^{0.06(v - E_K - 594)}}{1 + e^{-0.5(v - E_K + 5)}}. \tag{16}$$

Hyperpolarization activated funny current The formulation for the hyperpolarization activated funny current, I_f, is based on [4] and is given by

$$I_\mathrm{f} = g_\mathrm{f} o_\mathrm{f} (v - E_\mathrm{f}), \tag{17}$$

where

$$o_\mathrm{f} = x_\mathrm{f}, \tag{18}$$

and the dynamics of x_f is governed by an equation of the form (2).

L-type Ca^{2+} current The formualtion for the L-type Ca^{2+} current, I_CaL, is based on the formulation in [2] and is given by

$$I_\mathrm{CaL} = g_\mathrm{CaL} \left(Q_{10}^\mathrm{CaL}\right)^{Q_p} o_\mathrm{CaL} \frac{(2F)^2 v}{RT} \frac{0.341 c_d e^{\frac{2Fv}{RT}} - 0.341 c_e}{e^{\frac{2Fv}{RT}} - 1}, \tag{19}$$

where

$$o_\mathrm{CaL} = df(1 - f_\mathrm{Ca}), \tag{20}$$

and the dynamics of d, f and f_Ca are governed by equations of the form (2).

Background currents The formulation of the background currents, I_bCa and I_bCl, are based on [2] and are given by

$$I_\mathrm{bCa} = g_\mathrm{bCa}(v - E_\mathrm{Ca}), \tag{21}$$

$$I_\mathrm{bCl} = g_\mathrm{bCl}(v - E_\mathrm{Cl}). \tag{22}$$

Sodium-calcium exchanger The formulation of the Na^{+}-Ca^{2+} exchanger current, I_NaCa, is based on [2] and is given by

$$I_\mathrm{NaCa} = \bar{I}_\mathrm{NaCa} \left(Q_{10}^\mathrm{NaCa}\right)^{Q_p} \frac{e^{\frac{\nu F v}{RT}} [\mathrm{Na}^+]_i^3 c_e - e^{\frac{(\nu-1)Fv}{RT}} [\mathrm{Na}^+]_e^3 c_{sl}}{s_\mathrm{NaCa} \left(1 + \left(\frac{K_\mathrm{act}}{c_{sl}}\right)^2\right) \left(1 + k_\mathrm{sat} e^{\frac{(\nu-1)Fv}{RT}}\right)}, \tag{23}$$

where

$$s_\mathrm{NaCa} = K_{\mathrm{Ca},i} [\mathrm{Na}^+]_e^3 \left(1 + \left(\frac{[\mathrm{Na}^+]_i}{K_{\mathrm{Na},i}}\right)^3\right) + K_{\mathrm{Na},e}^3 c_{sl} \left(1 + \frac{c_{sl}}{K_{\mathrm{Ca},i}}\right) + K_{\mathrm{Ca},e} [\mathrm{Na}^+]_i^3 + [\mathrm{Na}^+]_i^3 c_e + [\mathrm{Na}^+]_e^3 c_{sl}.$$

Sarcolemmal Ca^{2+} pump The formulation of the current through the sarcolemmal Ca^{2+} pump, I_pCa, is based on [2] and is given by

$$I_\mathrm{pCa} = \bar{I}_\mathrm{pCa} \left(Q_{10}^\mathrm{pCa}\right)^{Q_p} \frac{c_{sl}^2}{K_\mathrm{pCa}^2 + c_{sl}^2}. \tag{24}$$

Sodium-potassium pump The current through the Na^+-K^+ pump, I_{NaK}, is based on [2] and is given by

$$I_{\mathrm{NaK}} = \bar{I}_{\mathrm{NaK}} \left(Q_{10}^{\mathrm{NaK}}\right)^{Q_p} \frac{f_{\mathrm{NaK}}}{1 + \left(\frac{K_{\mathrm{Na,i}}^{\mathrm{NaK}}}{[\mathrm{Na}^+]_{\mathrm{i}}}\right)^4} \frac{[\mathrm{K}^+]_{\mathrm{e}}}{[\mathrm{K}^+]_{\mathrm{e}} + K_{\mathrm{K,e}}}, \tag{25}$$

where

$$f_{\mathrm{NaK}} = \frac{1}{1 + 0.12 e^{-0.1 \frac{Fv}{RT}}} + \frac{0.037}{7} \left(e^{\frac{[\mathrm{Na}^+]_{\mathrm{e}}}{67}} - 1 \right) e^{-\frac{Fv}{RT}}. \tag{26}$$

Ca^{2+} dynamics

The Ca^{2+} dynamics are governed by

$$\frac{dc_d}{dt} = \frac{1}{V_d}(J_{\mathrm{CaL}} - J_d^b - J_d^c), \qquad \frac{db_d}{dt} = \frac{1}{V_d} J_d^b, \tag{27}$$

$$\frac{dc_{sl}}{dt} = \frac{1}{V_{sl}}(J_e^{sl} - J_{sl}^c - J_{sl}^b + J_s^{sl}), \qquad \frac{db_{sl}}{dt} = \frac{1}{V_{sl}} J_{sl}^b, \tag{28}$$

$$\frac{dc_c}{dt} = \frac{1}{V_c}(J_{sl}^c + J_d^c - J_c^n - J_c^b), \qquad \frac{db_c}{dt} = \frac{1}{V_c} J_c^b, \tag{29}$$

$$\frac{dc_s}{dt} = \frac{1}{V_s}(J_n^s - J_s^{sl} - J_s^b), \qquad \frac{db_s}{dt} = \frac{1}{V_s} J_s^b, \tag{30}$$

$$\frac{dc_n}{dt} = \frac{1}{V_n}(J_c^n - J_n^s). \tag{31}$$

Here, c_d is the concentration of free Ca^{2+} in the dyad, b_d is the concentration of Ca^{2+} bound to a buffer in the dyad, c_{sl} is the concentration of free Ca^{2+} in the sub-sarcolemmal (SL) compartment, b_{sl} is the concentration of Ca^{2+} bound to a buffer in the SL compartment, c_c is the concentration of free Ca^{2+} in the bulk cytosol, b_c is the concentration of Ca^{2+} bound to a buffer in the bulk cytosol, c_s is the concentration of free Ca^{2+} in the junctional sarcoplasmic reticulum (jSR), b_s is the concentration of Ca^{2+} bound to a buffer in the jSR, and c_n is the concentration of free Ca^{2+} in the network sarcoplasmic reticulum (nSR). The expressions for the fluxes are specified below.

Ca^{2+} fluxes

Flux through the SERCA pumps The flux from the bulk cytosol to the nSR through the SERCA pumps is based on [2] and given by

$$J_c^n = \bar{J}_{\text{SERCA}} \left(Q_{10}^{\text{SERCA}}\right)^{Q_p} \frac{\left(\frac{c_c}{K_c}\right)^2 - \left(\frac{c_n}{K_n}\right)^2}{1 + \left(\frac{c_c}{K_c}\right)^2 + \left(\frac{c_n}{K_n}\right)^2}. \tag{32}$$

Flux through the RyRs The flux from the jSR to the SL compartment is given by

$$J_s^{sl} = J_{\text{RyR}} + J_{\text{leak}}, \tag{33}$$

where J_{RyR} is the flux through the active RyR channels and J_{leak} is the flux through passive RyR channels that are always open, given by

$$J_{\text{RyR}} = p \cdot r \cdot \alpha_{\text{RyR}}(c_s - c_{sl}), \tag{34}$$

$$J_{\text{leak}} = \gamma_{\text{RyR}} \cdot \alpha_{\text{RyR}}(c_s - c_{sl}), \tag{35}$$

respectively. Here, p represents the open probability of the active RyR channels and is given by

$$p = \frac{c_d^3}{c_d^3 + \kappa_{\text{RyR}}^3}. \tag{36}$$

Furthermore, r is the fraction of RyR channels that are not inactivated and is governed by the equation

$$\frac{dr}{dt} = -\frac{J_{\text{RyR}}}{\beta_{\text{RyR}}} + \frac{\eta_{\text{RyR}}}{p}(1 - r). \tag{37}$$

Passive diffusion fluxes between compartments The passive diffusion fluxes between intracellular compartments are given by

$$J_d^c = \alpha_d^c(c_d - c_c), \tag{38}$$

$$J_{sl}^c = \alpha_{sl}^c(c_{sl} - c_c), \tag{39}$$

$$J_n^s = \alpha_n^s(c_n - c_s). \tag{40}$$

Buffer fluxes The fluxes of free Ca^{2+} binding to a Ca^{2+} buffer are given by

$$J_d^b = V_d(k_{\text{on}}^d c_d(B_{\text{tot}}^d - b_d) - k_{\text{off}}^d b_d), \tag{41}$$

$$J_{sl}^b = V_{sl}(k_{\text{on}}^{sl} c_{sl}(B_{\text{tot}}^{sl} - b_{sl}) - k_{\text{off}}^{sl} b_{sl}), \tag{42}$$

$$J_c^b = V_c(k_{\text{on}}^c c_c(B_{\text{tot}}^c - b_c) - k_{\text{off}}^c b_c), \tag{43}$$

$$J_s^b = V_s(k_{\text{on}}^s c_s(B_{\text{tot}}^s - b_s) - k_{\text{off}}^s b_s). \tag{44}$$

Membrane fluxes The membrane Ca^{2+} fluxes, J_{CaL}, J_{bCa}, J_{pCa}, and J_{NaCa}, are given by

$$J_{\text{CaL}} = -\frac{\chi C_m}{2F} I_{\text{CaL}}, \qquad J_{\text{pCa}} = -\frac{\chi C_m}{2F} I_{\text{pCa}}, \tag{45}$$

$$J_{\text{bCa}} = -\frac{\chi C_m}{2F} I_{\text{bCa}}, \qquad J_{\text{NaCa}} = \frac{\chi C_m}{F} I_{\text{NaCa}}, \tag{46}$$

where I_{CaL}, I_{bCa}, I_{pCa}, and I_{NaCa} are defined by the expressions given above. Furthermore,

$$J_e^{sl} = J_{\text{NaCa}} + J_{\text{pCa}} + J_{\text{bCa}}. \tag{47}$$

Na^+ dynamics

For the intracellular Na^+ concentration, we use a simple representation, ignoring possible concentration gradients between the intracellular compartments. In other words, we assume that the intracellular sodium concentration, $[Na^+]_i$, is constant throughout the cell and governed by the equation

$$\frac{d[\text{Na}_i]}{dt} = -\frac{\chi C_m}{F} \left(I_{\text{Na}} + I_{\text{NaL}} + 3I_{\text{NaK}} + 3I_{\text{NaCa}} + 0.3293 I_{\text{f}} \right), \tag{48}$$

where the currents I_{Na}, I_{NaL}, I_{NaK}, I_{NaCa}, and I_{f} are specified above. Here, the negative sign is due to the fact that the membrane currents are defined to be positive for a net flux of positive ions in the direction out of the cell. The factor χC_m converts the membrane currents from current per membrane capacitance to current per cell volume. Moreover, Faraday's constant, F, converts the currents from positive charges per time to mmoles of Na^+ ions per time.

The factor 3 in front of I_{NaK} is due to the fact that I_{NaK} pumps three Na^+ ions out of the cell and two K^+ ions in. Therefore, a net flow of one positive charge out of the cell through I_{NaK} corresponds to three Na^+ ions transported out of the cell. Similarly, I_{NaCa} exchanges three Na^+ against one Ca^{2+} ion, resulting in a decrease of three intracellular Na^+ ions for a net flow of one positive charge out of the cell.

The I_{f} current is composed of both Na^+ and K^+ ions. Based on [6, 7], the fraction of Na^+ transported by the current is assumed to be 0.3293.

Nernst equilibrium potentials

The Nernst equilibrium potentials for the ion channels are defined as

$$E_{\mathrm{Na}} = \frac{RT}{F}\log\left(\frac{[\mathrm{Na}^+]_{\mathrm{e}}}{[\mathrm{Na}^+]_{\mathrm{i}}}\right), \tag{49}$$

$$E_{\mathrm{Ca}} = \frac{RT}{2F}\log\left(\frac{[\mathrm{Ca}^{2+}]_{\mathrm{e}}}{c_{sl}}\right), \tag{50}$$

$$E_{\mathrm{K}} = \frac{RT}{F}\log\left(\frac{[\mathrm{K}^+]_{\mathrm{e}}}{[\mathrm{K}^+]_{\mathrm{i}}}\right), \tag{51}$$

$$E_{\mathrm{Ks}} = \frac{RT}{F}\log\left(\frac{[\mathrm{K}^+]_{\mathrm{e}} + 0.018[\mathrm{Na}^+]_{\mathrm{e}}}{[\mathrm{K}^+]_{\mathrm{i}} + 0.018[\mathrm{Na}^+]_{\mathrm{i}}}\right), \tag{52}$$

$$E_{\mathrm{Cl}} = \frac{RT}{F}\log\left(\frac{[\mathrm{Cl}^+]_{\mathrm{e}}}{[\mathrm{Cl}^+]_{\mathrm{i}}}\right), \tag{53}$$

$$E_{\mathrm{f}} = -17\ \mathrm{mV}, \tag{54}$$

for the parameter values given in Table II.

Parameter values

Parameter	Description	Value
V_d	Volume fraction of the dyadic subspace	0.001
V_{sl}	Volume fraction of the SL compartment	0.028
V_c	Volume fraction of the bulk cytosol	0.917
V_s	Volume fraction of the jSR	0.004
V_n	Volume fraction of the nSR	0.05
χ	Cell surface to volume ratio	0.6 μm^{-1} (adult)<br>0.9 μm^{-1} (hiPSC-CM)

Table I: Default geometry parameters of the base model.

Parameter	Description	Value
C_m	Specific membrane capacitance	0.01 μF/μm^2
F	Faraday's constant	96.485 C/mmol
R	Universal gas constant	8.314 J/(mol·K)
T	Temperature	310 K (adult)<br>296 K (hiPSC-CM)
$[Ca^{2+}]_e$	Extracellular Ca^{2+} concentration	1.8 mM
$[Na^{+}]_e$	Extracellular sodium concentration	140 mM (adult)<br>130 mM (hiPSC-CM)
$[K^{+}]_e$	Extracellular potassium concentration	5.4 mM
$[K^{+}]_i$	Intracellular potassium concentration	120 mM (adult)<br>180 mM (hiPSC-CM)
$[Cl^{-}]_e$	Extracellular chloride concentration	150 mM
$[Cl^{-}]_i$	Intracellular chloride concentration	15 mM

Table II: Physical constants and ionic concentrations of the base model.

Parameter	Value	Reference
Q_{10}^{Na}	2.0	[8, 3]
Q_{10}^{NaL}	2.2	[8, 3]
Q_{10}^{to}	2.0	[8]
$Q_{10}^{\text{Kr,act}}$	4.55	[8, 9]
$Q_{10}^{\text{Kr,inact}}$	3.08	[8, 9]
Q_{10}^{Ks}	2.0	[8]
Q_{10}^{f}	4.5	[8]
Q_{10}^{CaL}	1.8	[10]
Q_{10}^{NaCa}	1.6	[10]
Q_{10}^{pCa}	2.35	[10]
Q_{10}^{NaK}	1.6	[10]
Q_{10}^{KNaK}	1.5	[10]
Q_{10}^{SERCA}	2.6	[10]
Q^{p}	$\frac{T-310\text{ K}}{10\text{ K}}$	

Table III: Q_{10} values for the base model.

Parameter	Value (adult)	Value (hiPSC-CM)
g_{Na}	5.04 mS/μF	1.38 mS/μF
g_{NaL}	0.025 mS/μF	0.054 mS/μF
g_{to}	0.27 mS/μF	0.084 mS/μF
g_{Kr}	0.025 mS/μF	0.126 mS/μF
g_{Ks}	0.035 mS/μF	0.037 mS/μF
g_{K1}	0.37 mS/μF	0.045 mS/μF
g_{f}	0.0001 mS/μF	0.003 mS/μF
g_{bCl}	0.0102 mS/μF	0.0113 mS/μF
$\bar{I}_{\mathrm{NaK}}$	1.13 μA/μF	0.10 μA/μF
g_{CaL}	0.216 nL/(μF ms)	0.497 nL/(μF ms)
g_{bCa}	0.00039 mS/μF	0.000056 mS/μF
$\bar{I}_{\mathrm{NaCa}}$	4.9 μA/μF	13.5 μA/μF
$\bar{I}_{\mathrm{pCa}}$	0.34 μA/μF	0.77 μA/μF
$\bar{J}_{\mathrm{SERCA}}$	0.00024 mM/ms	0.00051 mM/ms
α_{RyR}	0.0075 ms^{-1}	0.0094 ms^{-1}
β_{RyR}	0.038 mM	0.0477 mM
α_d^c	0.0017 ms^{-1}	0.0044 ms^{-1}
α_{sl}^c	0.15 ms^{-1}	0.174 ms^{-1}
α_n^s	0.012 ms^{-1}	0.013 ms^{-1}
B_{tot}^c	0.07 mM	0.16 mM
B_{tot}^d	1.2 mM	4.36 mM
B_{tot}^{sl}	0.9 mM	2.23 mM
B_{tot}^s	27 mM	64.8 mM

Table IV: Cell-specific parameter values in the base model formulation

Parameter	Flux	Value
K_c	J_c^n	0.00025 mM
K_n	J_c^n	1.7 mM
γ_{RyR}	J_s^{sl}	0.001
κ_{RyR}	J_{RyR}	0.015 mM
η_{RyR}	J_s^{sl}	0.00001 ms^{-1}

Table V: Parameters for the intracellular Ca^{2+} fluxes of the base model.

Parameter	Current	Value
k_{sat}	I_{NaCa}	0.3
ν	I_{NaCa}	0.3
K_{act}	I_{NaCa}	0.00015 mM
$K_{\text{Ca},i}$	I_{NaCa}	0.0036 mM
$K_{\text{Ca},e}$	I_{NaCa}	1.3 mM
$K_{\text{Na},i}$	I_{NaCa}	12.3 mM
$K_{\text{Na},e}$	I_{NaCa}	87.5 mM
$K_{\text{Na},i}^{\text{NaK}}$	I_{NaK}	$(11\text{ mM})\cdot(Q_{10}^{\text{KNaK}})^{Q^p}$
$K_{\text{K},e}$	I_{NaK}	1.5 mM
K_{pCa}	I_{pCa}	0.0005 mM

Table VI: Additional parameters for the membrane currents of the base model.

Parameter	Compartment	Value
k_{on}^{c}	Bulk cytosol	40 $\text{ms}^{-1}\text{mM}^{-1}$
k_{off}^{c}	Bulk cytosol	0.03 ms^{-1}
k_{on}^{d}	Dyad	100 $\text{ms}^{-1}\text{mM}^{-1}$
k_{off}^{d}	Dyad	1 ms^{-1}
k_{on}^{sl}	Subsarcolemmal space	100 $\text{ms}^{-1}\text{mM}^{-1}$
k_{off}^{sl}	Subsarcolemmal space	0.15 ms^{-1}
k_{on}^{s}	Junctional SR	100 $\text{ms}^{-1}\text{mM}^{-1}$
k_{off}^{s}	Junctional SR	65 ms^{-1}

Table VII: Transition rates for the Ca^{2+} buffers of the base model.

Current	Gate	z_∞	α_z	β_z	τ_z
I_{Na}	m	$\frac{1}{(1+e^{(-57-v)/9})^2}$	$0.13e^{-((v+46)/16)^2}$	$0.06e^{-((v-5)/51)^2}$	$\frac{\alpha_m+\beta_m}{(Q_{10}^{\mathrm{Na}})^{Q_p}}$
	j	$\frac{1}{(1+e^{(v+72)/7})^2}$	0, if $v \geq -40$<br>$\frac{\begin{pmatrix} -2.5 \cdot 10^4 e^{0.2v} \\ -7 \cdot 10^{-6} e^{-0.04v} \end{pmatrix}(v+38)}{1+e^{0.3(v+79)}}$, otherwise	$\frac{0.6e^{0.06v}}{1+e^{-0.1(v+32)}}$, if $v \geq -40$<br>$\frac{0.02e^{-0.01v}}{1+e^{-0.14(v+40)}}$, otherwise	$\frac{1}{(\alpha_j+\beta_j)(Q_{10}^{\mathrm{Na}})^{Q_p}}$
I_{NaL}	m_L	$\frac{1}{1+e^{(-43-v)/5}}$	$\frac{1}{6.8e^{(v+12)/35}}$	$8.6e^{-(v+77)/6}$	$\frac{\alpha_m+\beta_m}{(Q_{10}^{\mathrm{NaL}})^{Q_p}}$
	h_L	$\frac{1}{1+e^{(v+88)/7.5}}$			$\frac{200 \text{ ms}}{(Q_{10}^{\mathrm{NaL}})^{Q_p}}$
I_{CaL}	d	$\frac{1}{1+e^{-(v+5)/6}}$	$\frac{1-e^{-\frac{v+5}{6}}}{0.035(v+5)}$		$\alpha_d d_\infty$
	f	$\frac{1}{1+e^{(v+35)/9}}+\frac{0.6}{1+e^{(50-v)/20}}$	$\frac{1}{0.02e^{-(0.034(v+14.5)^2)}+0.02}$		α_f
	f_{Ca}	$\frac{1.7c_d}{1.7c_d+0.012}$	$\frac{1}{1.7c_d+0.012}$		α_{Ca}
I_{to}	q_{to}	$\frac{1}{1+e^{(v+53)/13}}$	$\frac{39}{0.57e^{-0.08(v+44)}+0.065e^{0.1(v+46)}}$	6	$\frac{\alpha_{q_{\mathrm{to}}}+\beta_{q_{\mathrm{to}}}}{(Q_{10}^{\mathrm{to}})^{Q_p}}$
	r_{to}	$\frac{1}{1+e^{-(v-22.3)/18.75}}$	$\frac{14.4}{e^{0.09(v+30.61)}+0.37e^{-0.12(v+24)}}$	2.75	$\frac{\alpha_{r_{\mathrm{to}}}+\beta_{r_{\mathrm{to}}}}{(Q_{10}^{\mathrm{to}})^{Q_p}}$
I_{Kr}	x_{Kr1}	$\frac{1}{1+e^{-(v-2.7)/15.3}}$	$\frac{450}{1+e^{-(v+45)/10}}$	$\frac{6}{1+e^{(v+30)/11.5}}$	$\frac{\alpha_{x_{\mathrm{Kr1}}}\cdot\beta_{x_{\mathrm{Kr1}}}}{(Q_{10}^{\mathrm{Kr,act}})^{Q_p}}$
	x_{Kr2} (WT)	$\frac{1}{1+e^{(v+70)/20.9}}$	$\frac{3}{1+e^{-(v+60)/20}}$	$\frac{1.12}{1+e^{(v-60)/20}}$	$\frac{\alpha_{x_{\mathrm{Kr2}}}\cdot\beta_{x_{\mathrm{Kr2}}}}{(Q_{10}^{\mathrm{Kr,inact}})^{Q_p}}$
	x_{Kr2} (SQT)	$\frac{1}{1+e^{(v-70-62)/(20.9\cdot 1.85)}}$	$\frac{3}{1+e^{-(v+60)/20}}$	$\frac{1.12}{1+e^{(v-60)/20}}$	$\frac{\alpha_{x_{\mathrm{Kr2}}}\cdot\beta_{x_{\mathrm{Kr2}}}}{(Q_{10}^{\mathrm{Kr,inact}})^{Q_p}}$
I_{Ks}	x_{Ks}	$\frac{1}{1+e^{-(v+3.8)/14}}$	$\frac{990}{1+e^{-(v+2.4)/14}}$		$\frac{\alpha_{x_{\mathrm{Ks}}}}{(Q_{10}^{\mathrm{Ks}})^{Q_p}}$
I_{f}	x_{f}	$\frac{1}{1+e^{(v+78)/5}}$	$\frac{1900}{1+e^{(v+15)/10}}$		$\frac{\alpha_{x_{\mathrm{f}}}}{(Q_{10}^{\mathrm{f}})^{Q_p}}$

Table VIII: Specification of the parameters z_∞ and τ_z, for $z = m$, j, m_L, h_L, d, f, f_{Ca}, q_{to}, r_{to}, x_{Kr1}, x_{Kr2}, x_{Ks} and x_{f} in the equations for the gating variables (2).

References

[1] Karoline Horgmo Jæger, Verena Charwat, Bérénice Charrez, Henrik Finsberg, Mary M Maleckar, Sam Wall, Kevin Healy, and Aslak Tveito. Improved computational identification of drug response using optical measurements of human stem cell derived cardiomyocytes in microphysiological systems. *Frontiers in Pharmacology*, 10:1648, 2020.

[2] Eleonora Grandi, Francesco S Pasqualini, and Donald M Bers. A novel computational model of the human ventricular action potential and Ca transient. *Journal of Molecular and Cellular Cardiology*, 48(1):112–121, 2010.

[3] Thomas O'Hara, László Virág, András Varró, and Yoram Rudy. Simulation of the undiseased human cardiac ventricular action potential: Model formulation and experimental validation. *PLoS Computational Biology*, 7(5):e1002061, 2011.

[4] Michelangelo Paci, Jari Hyttinen, Katriina Aalto-Setälä, and Stefano Severi. Computational models of ventricular-and atrial-like human induced pluripotent stem cell derived cardiomyocytes. *Annals of Biomedical Engineering*, 41(11):2334–2348, 2013.

[5] Ibrahim El-Battrawy, Huan Lan, Lukas Cyganek, Zhihan Zhao, Xin Li, Fanis Buljubasic, Siegfried Lang, Gökhan Yücel, Katherine Sattler, Wolfram-Hubertus Zimmermann, et al. Modeling short QT syndrome using human-induced pluripotent stem cell–derived cardiomyocytes. *Journal of the American Heart Association*, 7(7):e007394, 2018.

[6] Divya C Kernik, Stefano Morotti, HaoDi Wu, Priyanka Garg, Henry J Duff, Junko Kurokawa, José Jalife, Joseph C Wu, Eleonora Grandi, and Colleen E Clancy. A computational model of induced pluripotent stem-cell derived cardiomyocytes incorporating experimental variability from multiple data sources. *The Journal of Physiology*, 2019.

[7] Arie O Verkerk and Ronald Wilders. Hyperpolarization-activated current, in mathematical models of rabbit sinoatrial node pacemaker cells. *BioMed Research International*, 2013, 2013.

[8] Michelangelo Paci, Elisa Passini, Aleksandra Klimas, Stefano Severi, Jari Hyttinen, Blanca Rodriguez, and Emilia Entcheva. *In silico* populations optimized on optogenetic recordings predict drug effects in human

induced pluripotent stem cell-derived cardiomyocytes. In *2018 Computing in Cardiology Conference (CinC)*, volume 45, pages 1–4. IEEE, 2018.

[9] Maike Mauerhöfer and Christiane K Bauer. Effects of temperature on heteromeric Kv11. 1a/1b and Kv11. 3 channels. *Biophysical Journal*, 111(3):504–523, 2016.

[10] Thomas R Shannon, Fei Wang, José Puglisi, Christopher Weber, and Donald M Bers. A mathematical treatment of integrated Ca dynamics within the ventricular myocyte. *Biophysical Journal*, 87(5):3351–3371, 2004.
