# Supplementary material for: Computational prediction of drug response in short QT syndrome type 1 based on measurements of compound effect in stem cell-derived cardiomyocytes
Source: PLoS Comput Biol. 2021 Feb 16;17(2):e1008089. doi: 10.1371/journal.pcbi.1008089 (PMC7909705; doi:10.1371/journal.pcbi.1008089)
Supplement: S2 Appendix — (PDF) [file pcbi.1008089.s002.pdf]

A note on the sign of the pseudo-ECG

In the pseudo-ECG computed using the method described in the Methods section of the paper, we observed that the T-wave had the opposite sign for the SQT1 case compared to the WT case (see Fig 6 in the paper). In this appendix, we give a simplified explanation of why the T-wave is negative in the pseudo-ECG computed for the SQT1 case. Note, however, that the change of sign of the T-wave is not a known feature of SQT1 syndrome (see, e.g., the ECGs of patients with SQT1 in Figure 1 of [1] and Figure 1 of [2]), so this change in the T-wave is likely just a consequence of our simplified pseudo-ECG computations.

Pseudo-ECG computations

The pseudo-ECG is computed using the cable equation for a strand of connected cells, representing an endocardial, a midmyocardial and an epicardial region. The extracellular potential, u_e, making up the ECG is recorded in a point, r, located 2 cm to the right of the end of the epicardial region (see the upper panel of Fig 1). The extracellular potential is computed by the formula

$$u_e = \frac{1}{4\pi\sigma_e} \sum_k \frac{I_m^k}{|r - r_k|}, \tag{1}$$

where σ_e is the extracellular conductivity, $|r - r_k|$ is the distance between the measurement point r and the center of each of the cells k in the cell strand, and I_m^k are the membrane currents from each of the cells in the cell strand. Assuming an homogeneous intracellular conductivity, σ_i, this I_m is given by a numerical approximation of

$$I_m = \delta \frac{\partial^2 v}{\partial x^2}, \tag{2}$$

where v is the membrane potential and δ is a constant (see the Methods section of the paper).

Depolarization wave

The pseudo-ECG computation is initiated by depolarizing the membrane potential of the first two (leftmost) cells of the endocardial region. This generates a propagating depolarization wave, travelling in the direction from left to right. This wave is similar in the WT and SQT1 cases (see Fig 1). In the upper panels of the plots in Fig 1, we have plotted an example traveling wave, where v is given by a $\tanh(x)$-function. In the panels below, we have plotted the double derivative of this function, which is proportional to I_m (see (2)). From the formula (1), we observe that in the computation of the pseudo-ECG, the membrane currents I_m are divided by the distance from the measuring point, r. Therefore, membrane currents on the right part of the domain will contribute more to the pseudo-ECG than membrane currents on the left part of the domain. Furthermore, in (1), we observe that u_e will have the same sign as $\sum_k \frac{I_m^k}{|r-r_k|}$. Since I_m is positive on the right-hand side during the depolarization wave, we thus get a positive signal (QRS-complex) in the pseudo ECG during the depolarization wave.

Repolarization wave

After the cells have been depolarized for a while, they repolarize to the resting state. If the properties of the cells were similar everywhere, it would be natural for repolarization to first occur in the endocardial region, since these cells were depolarized first. However, the repolarizing currents I_{to} and I_{Ks} are stronger in the epicardial than in the endocardial and midmyocardial regions (see the upper panel of Fig 1). In the normal WT case, the increased strength of these currents outweighs the fact that the epicardial cells were depolarized last. Therefore, the repolarization wave starts in the epicardial region and moves towards the endocardial region. Example shapes of the repolarization wave and the corresponding I_m are plotted in Fig 1. We observe that I_m is again positive on the right-hand side, leading to a positive T-wave.

In the SQT1 case, on the other hand, the I_{Kr} current has increased strength in all cells and is therefore more important for the repolarization of the cells. As a result, the increased strength of I_{to} and I_{Ks} in the epicardial region is less important and no longer outweighs the fact that that the endocardial cells are depolarized first. Repolarization therefore occurs in the endocardial region first and moves towards the epicardial region. The shape of the wave is now the opposite of the shape of the wave in the WT case, and I_m is negative on the right-hand side, explaining why the T-wave is negative in the pseudo-ECG in the SQT1 case.

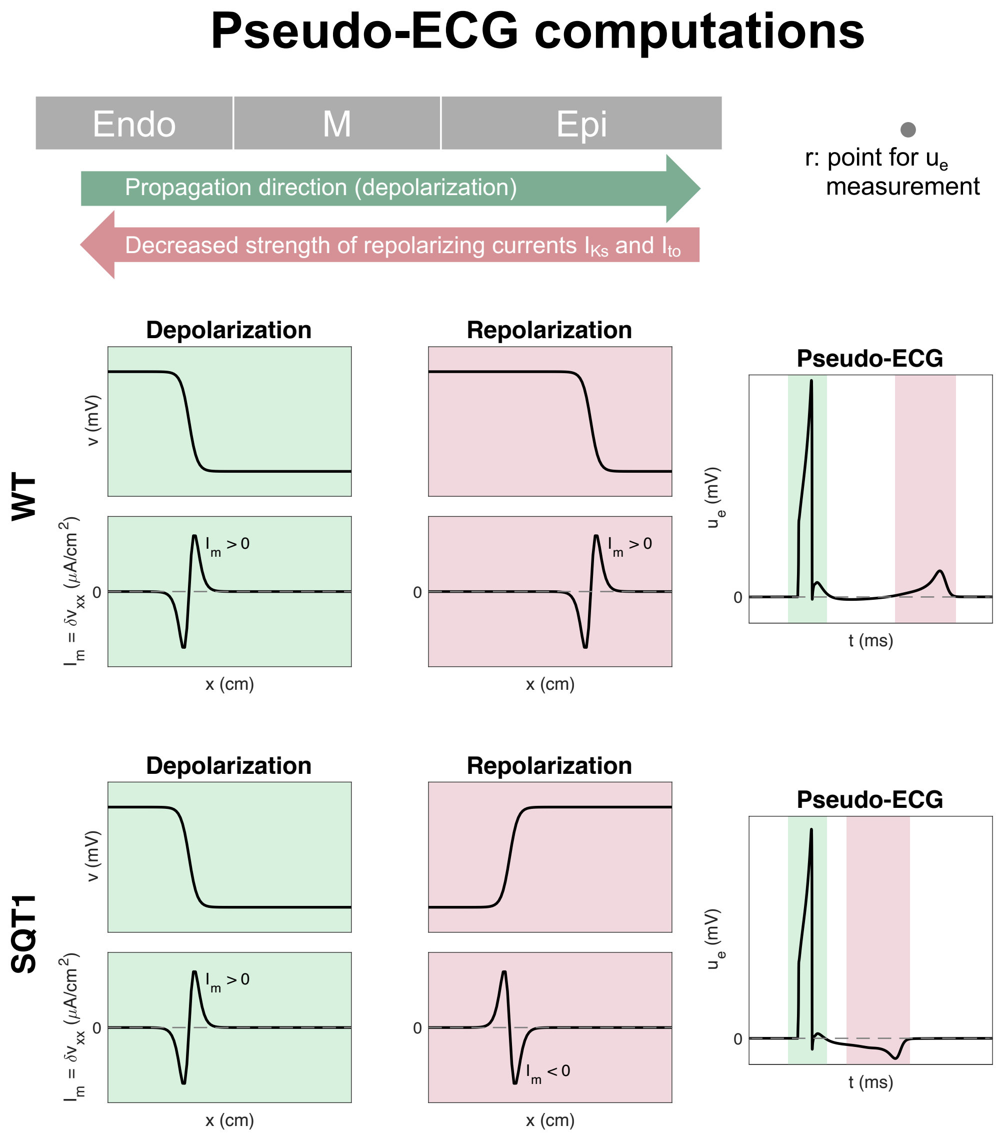

Fig 1: Illustration of the mechanism underlying the change of sign of the T-wave in the pseudo-ECG computed for the SQT1 case. An explanation is given in the text of this appendix. Note here that the plots in the right panel (the pseudo-ECGs) are taken directly from the pseudo-ECG simulations in Fig 6 of the paper, while the plots in the left and middle panels are simplified representations of the propagating wave.

References

1. El-Battrawy I, Lan H, Cyganek L, Zhao Z, Li X, Buljubasic F, et al. Modeling Short QT Syndrome Using Human-Induced Pluripotent Stem Cell–Derived Cardiomyocytes. Journal of the American Heart Association. 2018;7(7):e007394.

2. Wolpert C, Schimpf R, Giustetto C, Antzelevitch C, Cordeiro J, Dumaine R, et al. Further insights into the effect of quinidine in short QT syndrome caused by a mutation in HERG. Journal of Cardiovascular Electrophysiology. 2005;16(1):54–58.
