# Supplementary figures and images for: Computational prediction of drug response in short QT syndrome type 1 based on measurements of compound effect in stem cell-derived cardiomyocytes
Source: PLoS Comput Biol. 2021 Feb 16;17(2):e1008089. doi: 10.1371/journal.pcbi.1008089 (PMC7909705; doi:10.1371/journal.pcbi.1008089)

**RMP**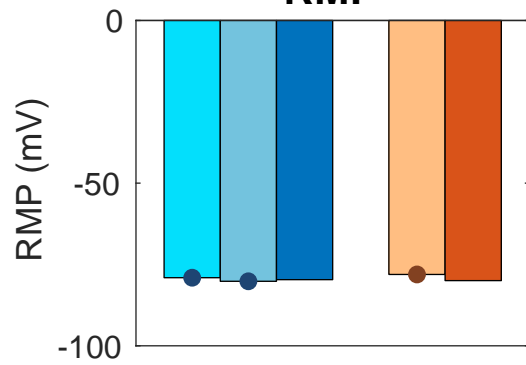**APA**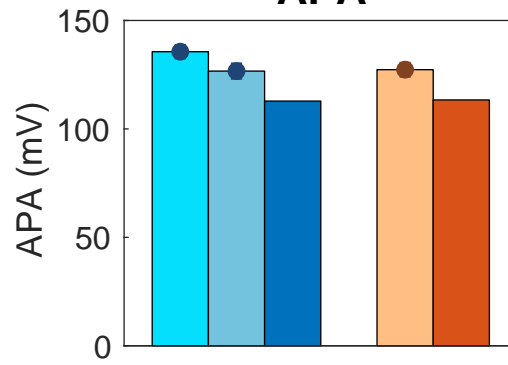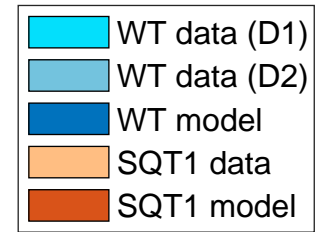**dvd<sub>t</sub><sub>max</sub>**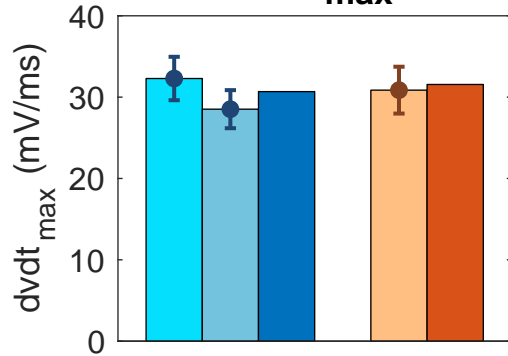**APD50**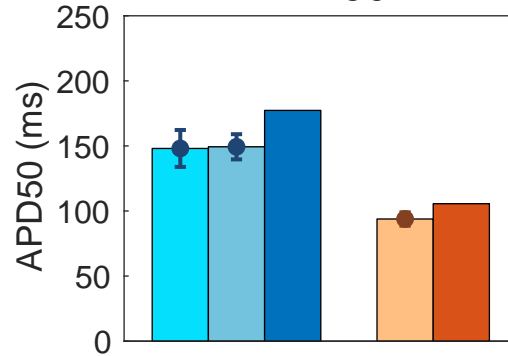**APD90**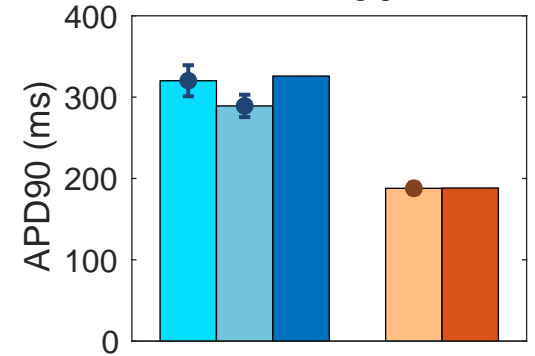**I<sub>Ks</sub>**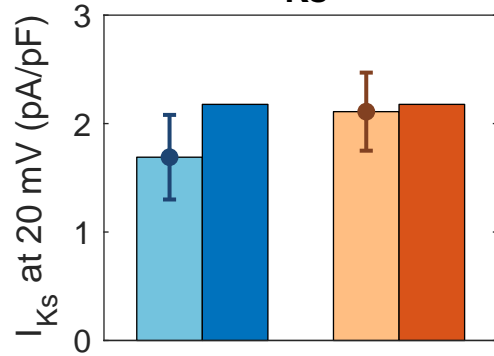**I<sub>to</sub>**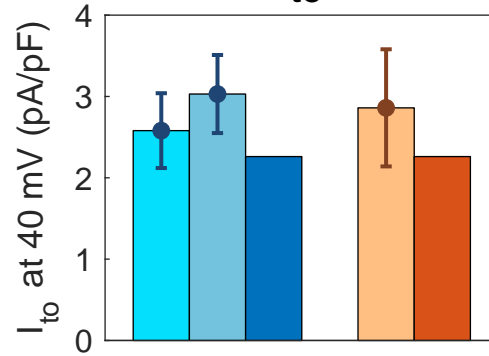**I<sub>K1</sub>**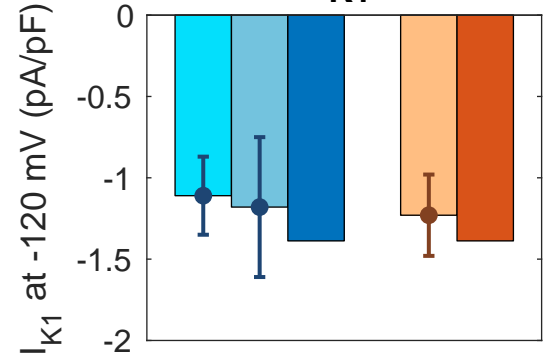**I<sub>CaL</sub>**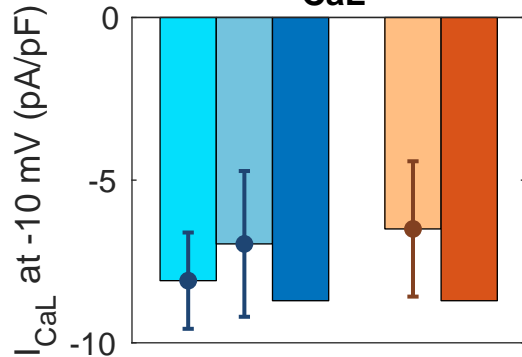**I<sub>NaL</sub>**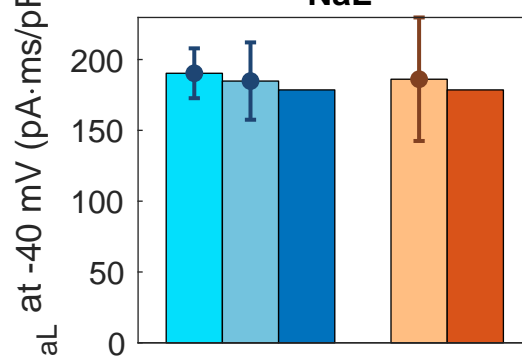

Supplement: S1 Fig — Comparison of data from [9] for WT and SQT1 hiPSC-CMs to properties of the hiPSC-CM base model for WT and SQT1 used as a starting point for the inversions. The upper two rows compare the resting membrane potential (RMP), action potential amplitude (APA), maximal upstroke velocity (dvdtmax) and action potential durations at 50% and 90% repolarization (APD50 and APD90). The lower two rows compare the current densities of IKs, Ito, IK1, ICaL and INaL at specific voltages using the voltage clamp procedures described in [9]. Note that two different WT hiPSC lines (D1 and D2) were considered in [9]. Note also that the IKr current reported in [9] was not compared to the model because the measurements of IKr in [9] were conducted using Cs+ ions instead of K+ ions, which could influence the properties of the current, making the measurements less appropriate for direct comparison to the model current [9, 89]. (PDF) [file pcbi.1008089.s005.pdf]

## Action potential

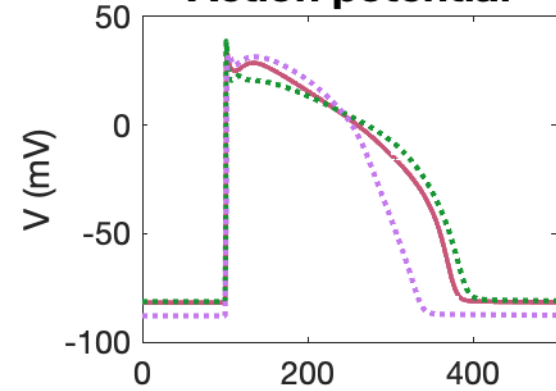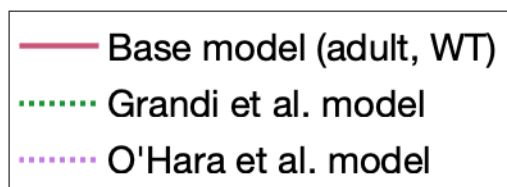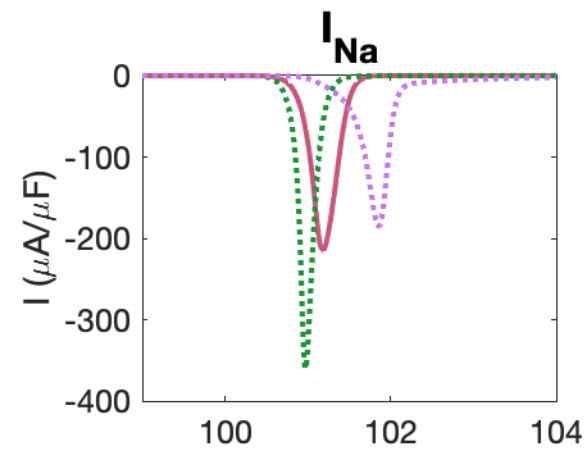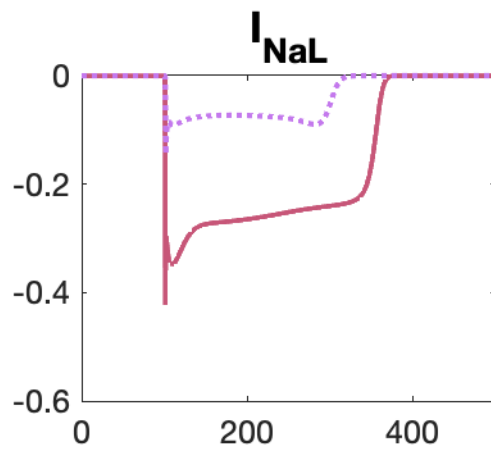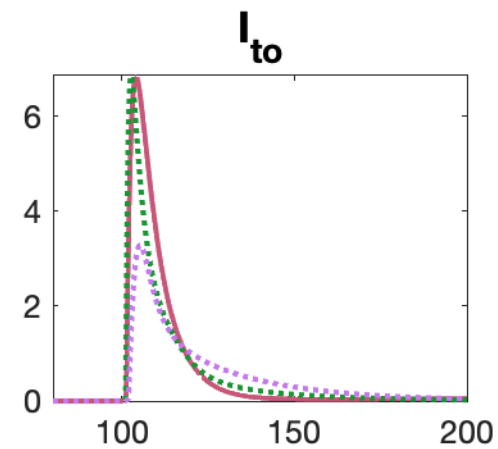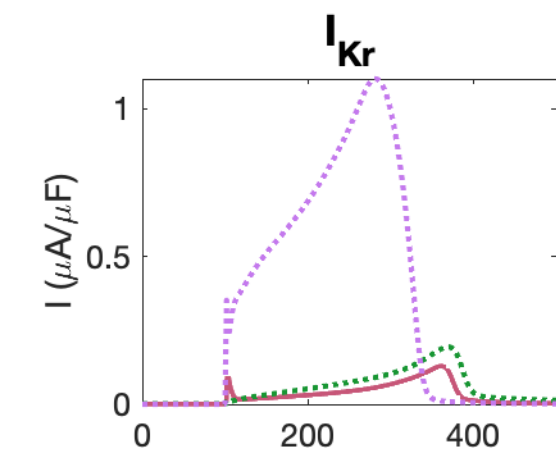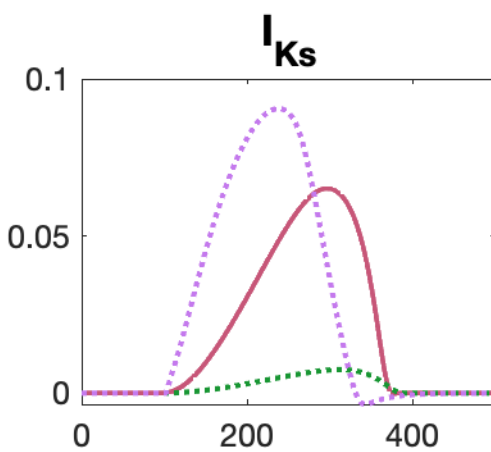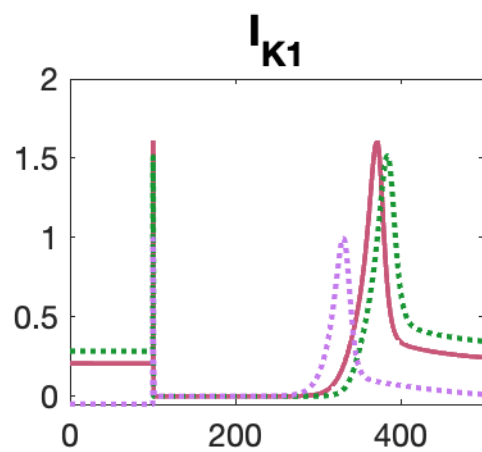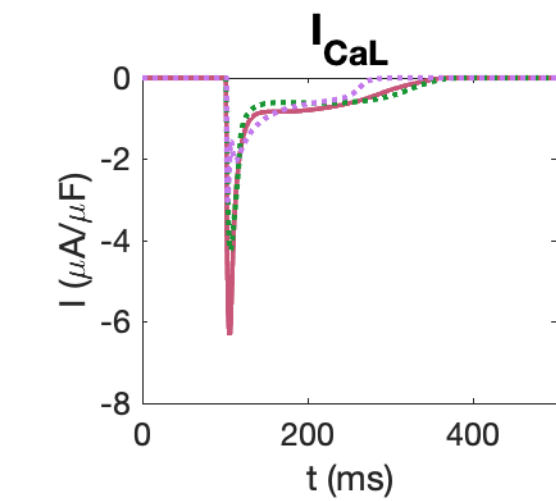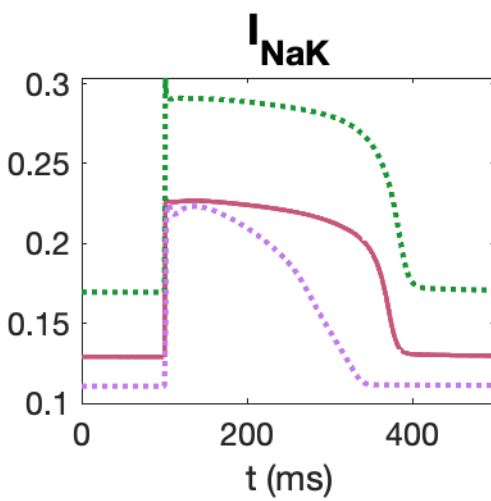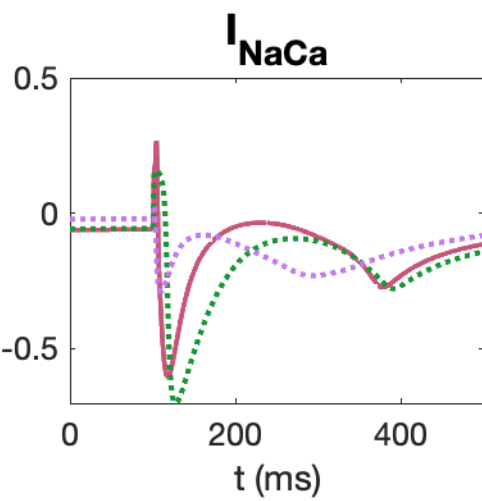

Supplement: S2 Fig — Comparison of the action potential and some of the main currents in the adult WT base model used in this study against the action potential and currents from the Grandi et al. and O’Hara et al. models [27, 90]. The currents and action potentials are computed in a simulation using 1 Hz pacing, and the epicardial version of the models are used for all three models. (PDF) [file pcbi.1008089.s006.pdf]

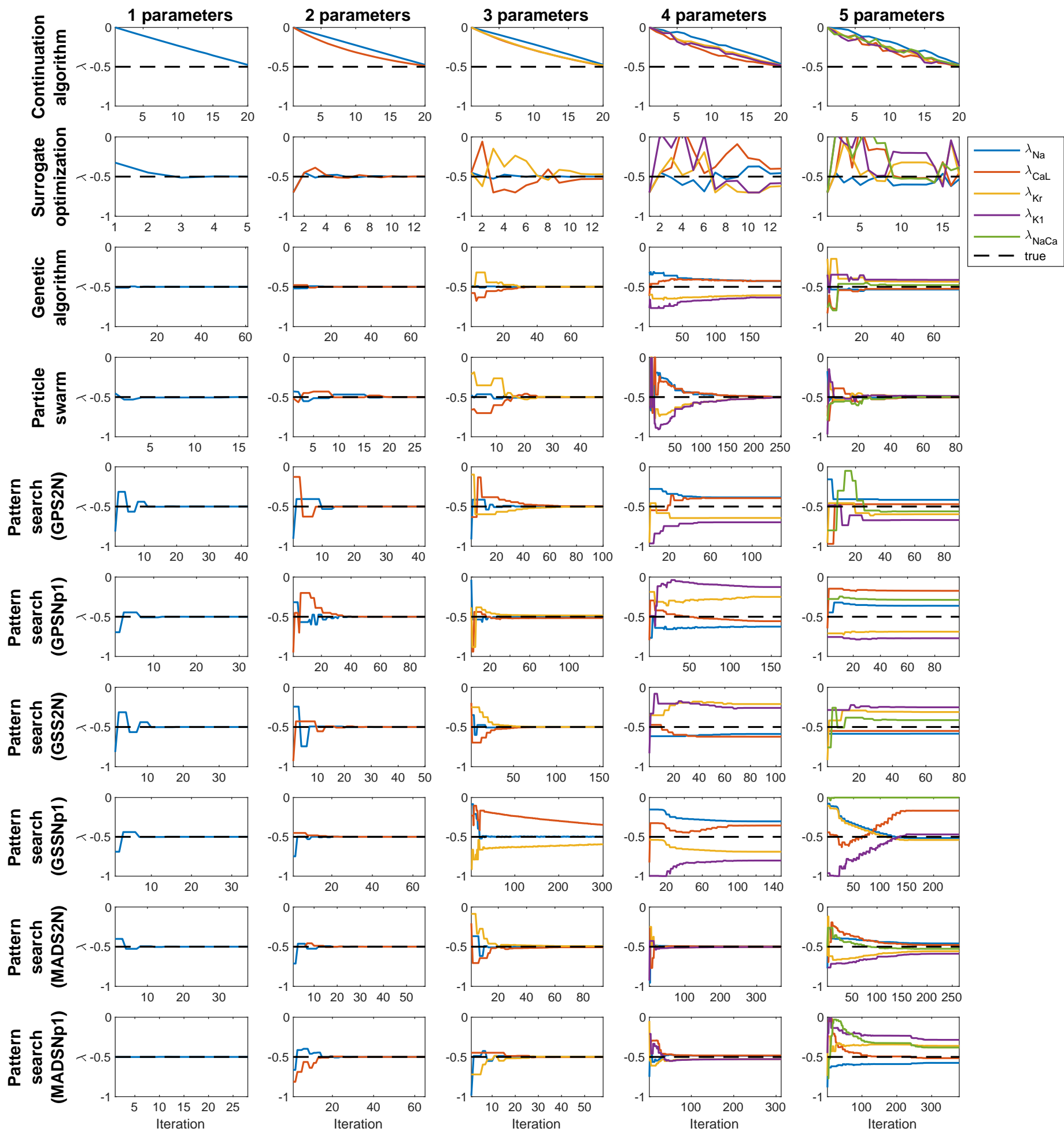

Supplement: S4 Fig — Comparison of the continuation method and a number of other optimization methods from MATLAB’s Global Optimization Toolbox [45]. We use the same cost function as in the inversions of the paper, except that we include no regularization terms and only consider a single AP (not several doses). The data is defined by simulated solutions generated using λ = −0.5 for all considered currents (see the legends). The titles above each column specify the considered number of free parameters, and the plots show the evolution of the optimal parameter values during the iterations of the algorithms. We impose the bounds −1 ≤ λ ≤ 0 and a maximum time limit of 5 hours for all methods, except that the bounds are adjusted to −0.7 ≤ λ ≤ 0.3 for the surrogate optimization algorithm, to avoid automatically finding the correct solution, λ = −0.5, in the first iteration of the algorithm. (PDF) [file pcbi.1008089.s008.pdf]
