# Supplementary material for: Computational prediction of drug response in short QT syndrome type 1 based on measurements of compound effect in stem cell-derived cardiomyocytes
Source: PLoS Comput Biol. 2021 Feb 16;17(2):e1008089. doi: 10.1371/journal.pcbi.1008089 (PMC7909705; doi:10.1371/journal.pcbi.1008089)
Supplement: S3 Fig — Singular value decomposition analysis of the SQT1 hiPSC-CM base model, using the method from [73]. The identifiability of each current is indicated in the orange panel, and a value close to 1 indicates a high degree of identifiability. The cost function used to define the unidentifiable space is the same as that used in the inversions. The identifiability threshold, δ, was set to 0.1. For details on the analysis, we refer to [73]. (PDF) [file pcbi.1008089.s007.pdf]

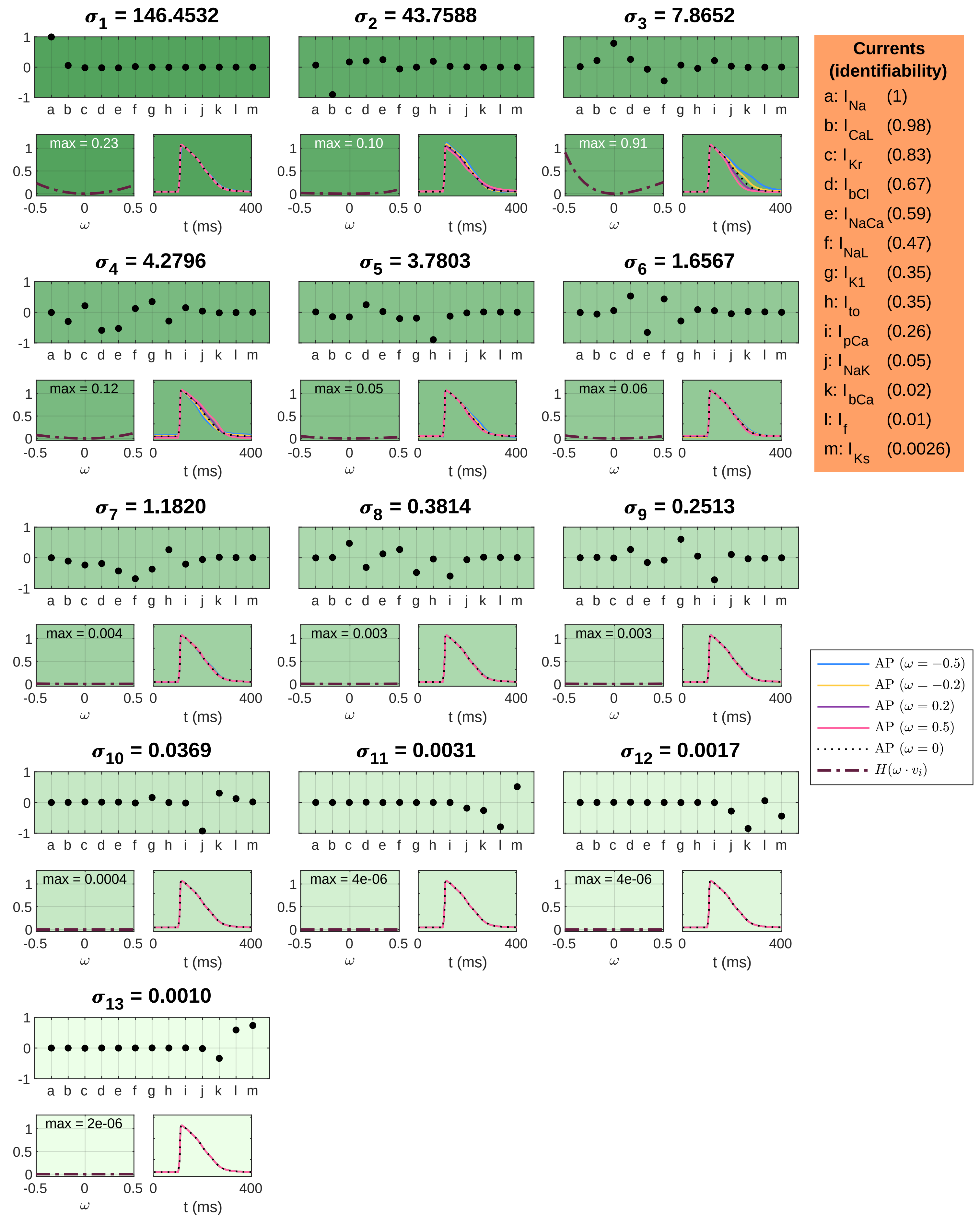
σ1 = 146.4532
σ2 = 43.7588
σ3 = 7.8652
σ4 = 4.2796
σ5 = 3.7803
σ6 = 1.6567
σ7 = 1.1820
σ8 = 0.3814
σ9 = 0.2513
σ10 = 0.0369
σ11 = 0.0031
σ12 = 0.0017
σ13 = 0.0010
max = 0.23
max = 0.10
max = 0.91
max = 0.12
max = 0.05
max = 0.06
max = 0.004
max = 0.003
max = 0.003
max = 0.0004
max = 4e-06
max = 4e-06
max = 2e-06
a b c d e f g h i j k l m
ω
t (ms)
Currents (identifiability)
a: I_Na (1)
b: I_CaL (0.98)
c: I_Kr (0.83)
d: I_bCl (0.67)
e: I_NaCa (0.59)
f: I_NaL (0.47)
g: I_K1 (0.35)
h: I_to (0.35)
i: I_pCa (0.26)
j: I_NaK (0.05)
k: I_bCa (0.02)
l: I_f (0.01)
m: I_Ks (0.0026)
AP (ω = −0.5)
AP (ω = −0.2)
AP (ω = 0.2)
AP (ω = 0.5)
AP (ω = 0)
H(ω · v_i)
